# Antiviral, Antifungal and Antibacterial Activities of a BODIPY-Based Photosensitizer

**DOI:** 10.3390/molecules200610604

**Published:** 2015-06-08

**Authors:** Bradley L. Carpenter, Xingci Situ, Frank Scholle, Juergen Bartelmess, Walter W. Weare, Reza A. Ghiladi

**Affiliations:** 1 Department of Chemistry, North Carolina State University, Raleigh, NC 27695-8204, USA; E-Mails: blcarpen@ncsu.edu (B.L.C.); xsitu@ncsu.edu (X.S.); Juergen.Bartelmess@iit.it (J.B.); wwweare@ncsu.edu (W.W.W.); 2 Department of Biological Sciences, North Carolina State University, Raleigh, NC 27695-7614, USA; E-Mail: fscholl@ncsu.edu

**Keywords:** photodynamic therapy, singlet oxygen, antibacterial, antiviral, antifungal, photobiocidal

## Abstract

Antimicrobial photodynamic inactivation (aPDI) employing the BODIPY-based photosensitizer 2,6-diiodo-1,3,5,7-tetramethyl-8-(*N*-methyl-4-pyridyl)-4,4′-difluoro-boradiazaindacene (DIMPy-BODIPY) was explored in an *in vitro* assay against six species of bacteria (eight total strains), three species of yeast, and three viruses as a complementary approach to their current drug-based or non-existent treatments. Our best results achieved a noteworthy 5–6 log unit reduction in CFU at 0.1 μM for *Staphylococcus aureus* (ATCC-2913), methicillin-resistant *S. aureus* (ATCC-44), and vancomycin-resistant *Enterococcus faecium* (ATCC-2320), a 4–5 log unit reduction for *Acinetobacter baumannii* ATCC-19606 (0.25 μM), multidrug resistant *A. baumannii* ATCC-1605 (0.1 μM), *Pseudomonas aeruginosa* ATCC-97 (0.5 μM), and *Klebsiella pneumoniae* ATCC-2146 (1 μM), and a 3 log unit reduction for *Mycobacterium smegmatis* mc^2^155 (ATCC-700084). A 5 log unit reduction in CFU was observed for *Candida albicans* ATCC-90028 (1 μM) and *Cryptococcus neoformans* ATCC-64538 (0.5 μM), and a 3 log unit reduction was noted for *Candida glabrata* ATCC-15545 (1 μM). Infectivity was reduced by 6 log units in dengue 1 (0.1 μM), by 5 log units (0.5 μM) in vesicular stomatitis virus, and by 2 log units (5 μM) in human adenovirus-5. Overall, the results demonstrate that DIMPy-BODIPY exhibits antiviral, antibacterial and antifungal photodynamic inactivation at nanomolar concentrations and short illumination times.

## 1. Introduction

Despite the advent of antibiotics and vaccines, infectious diseases remain the leading worldwide cause of mortality and morbidity [1]. Accounting for over 60% of deaths in the developing world, they are also the third and fourth leading causes of death in Europe and the United States, respectively [1,2]. Efforts to control microbial infections have been hampered by the emergence and proliferation of drug resistant pathogens, necessitating the pursuit of complementary approaches to the current drug-based treatments. Furthermore, a scarcity of effective therapies for many globally important viral infections stresses the need for development of novel approaches for either treatment or prevention of infection. One such option, antimicrobial photodynamic inactivation (aPDI), is currently being explored as a potential therapeutic treatment option for various types of infection, whether bacterial, fungal, viral, or even parasitic in nature [3,4,5,6,7]. aPDI makes use of a photosensitizer (PS) to generate reactive oxygen species (*i.e.*, radicals or singlet oxygen (^1^O_2_)) upon illumination with light (visible or near infrared). Although challenges with aPDI exist, such as issues of tissue penetration with light and photosensitizer selectivity, as a biocidal agent, ^1^O_2_ possesses a number of unique properties that make it particularly attractive for antimicrobial applications. These include damaging reactivity with most biomolecules, a short lifetime of ~10^−6^ s in aqueous environments, and the formation of harmless ground state molecular oxygen if left unreacted [8,9]. More importantly, development of bacterial resistance to aPDI is believed to be unlikely due to the non-specific damage caused by ^1^O_2_ [10]. Additionally, a number of studies have shown that a photodynamic inactivation strategy is equally effective against both drug-susceptible and drug-resistant bacterial strains, demonstrating the enormous potential of aPDI in combating pathogenic infections [11,12].

**Figure 1:**
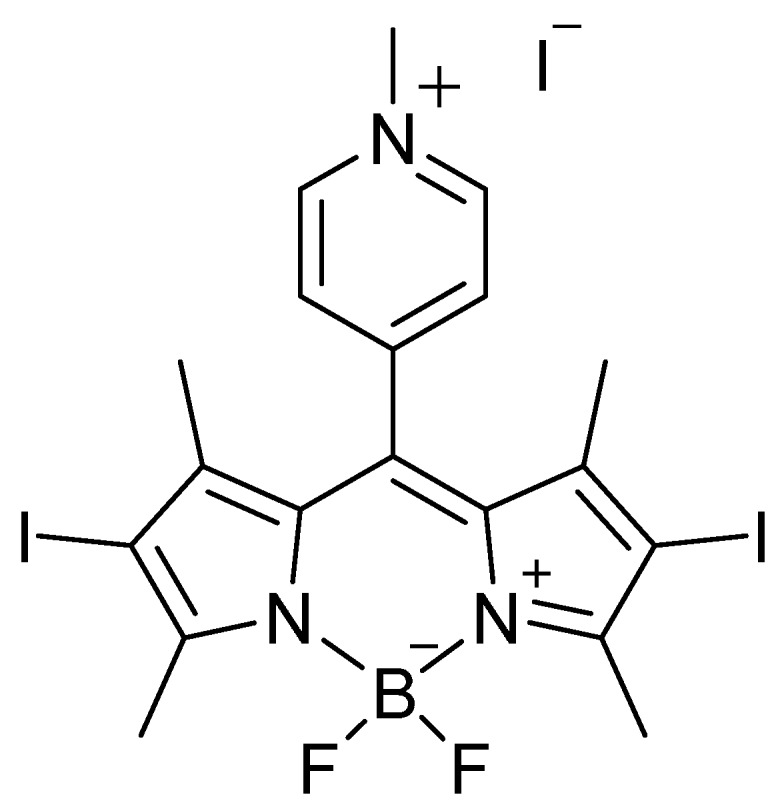
The DIMPy-BODIPY photosensitizer employed in this study.

While numerous efforts have focused on photosensitizer scaffolds based upon cationic tetrapyrrole-related macrocycles (porphyrins, bacteriochlorins, phthalocyanines) or other conjugated systems [13,14,15,16], in particular methylene blue [17,18,19,20], very little is known regarding the applicability of boron dipyrromethene (a.k.a. BODIPY)-based compounds as potential photosensitizers for aPDI. BODIPY dyes exhibit a number of properties that are potentially attractive for photodynamic applications, including synthetic tunability of their absorption features, chemical robustness, and excellent hydrolytic stability under physiological conditions [21,22,23,24,25]. Of particular interest for our work are two recent studies: The first, by Caruso *et al.* [26], investigated two novel cationic and iodinated BODIPYs as photosensitizers, but only against two bacterial model strains, *Escherichia coli* and *Staphylococcus xylosus*. The second study, by O’Shea *et al.* [27], examined the aPDI application of the structurally-related aza-BODIPY photosensitizers against *E. coli*, *S. aureus*, and *Candida albicans*. Outside of these two studies, reports of a BODIPY-based photosensitizer employed in antimicrobial photodynamic inactivation are limited, and to the best of our knowledge no studies have been performed against viruses with this class of photosensitizer. Given that the majority of nosocomial infections in the United States are caused by *Staphylococcus aureus* (15%), enterococci species (12%), candida species (11%), *Pseudomonas aeruginosa* (8%), *Klebsiella pneumoniae* (6%), enterobacter species (5%) and *Acinetobacter baumannii* (3%) [28,29], the fact that relatively few clinically-relevant pathogenic bacterial and fungal species, and importantly no viruses, have been explored with BODIPY-based systems highlights a lack of understanding of this new class of photosensitizer with pathogens of direct importance to human health and disease. Herein, to further investigations into the feasibility of aPDI for future therapeutic use as a complementary approach to drug-based treatments, we explored the extent of *in vitro* antimicrobial photodynamic inactivation employing the DIMPy-BODIPY [26,30] photosensitizer (Figure 1) against eight species of bacteria and three species of yeast that together fall within the five classes of antibiotic-resistant pathogens that are emerging as major public health threats: vancomycin-resistant enterococci (VRE), methicillin-resistant *Staphylococcus aureus* (MRSA), multidrug-resistant mycobacteria, Gram-negative bacteria, and fungi [31]. We also extended our study to include dengue-1 virus, vesicular stomatitis virus (VSV), and human adenovirus-5 as model DNA viruses to explore the potential of antiviral PDI [32,33,34,35] using BODIPY-based photosensitizers for non-*in vivo* surface sterilization or materials applications.

## 2. Results and Discussion

### 2.1. Antiviral Photodynamic Inactivation Studies

**Figure 2:**
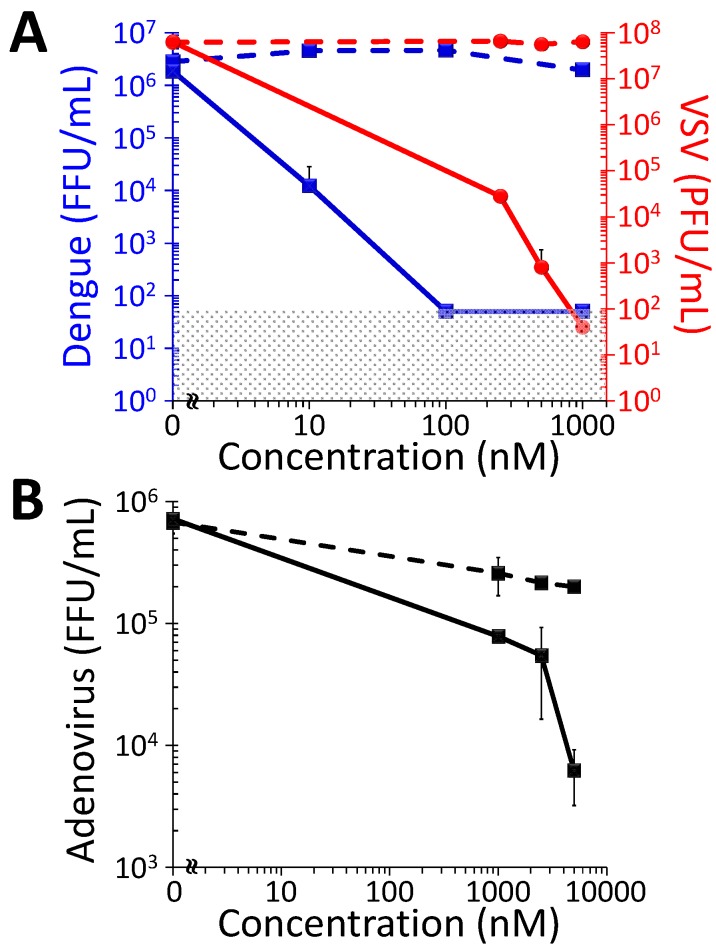
Photodynamic inactivation as a function of DIMPy-BODIPY concentration of (**A**) dengue 1 virus (blue) and vesicular stomatitis virus (VSV, red); and (**B**) human adenovirus-5 (HAd-5). In both panels, the solid lines represent the light treated samples, whereas the dashed lines represent the dark controls. For these studies, the illumination conditions were as follows: 30 min, 400–700 nm, 65 ± 5 mW/cm^2^ (total fluence of 118 J/cm^2^). The detection limits of the assays were 50 FFU/mL for the dengue virus study, 40 PFU/mL for the VSV study, and 66 FFU/mL for the HAd-5 study; data points below the detection limit were set to these values (represented by the shaded area) for graphing purposes. In the cases where error bars cannot be visualized, the error bars themselves were smaller than the marker employed in the plot.

The potential efficacy of DIMPy-BODIPY as a photodynamic inactivator of viral agents was tested using three viruses from different families: The enveloped viruses dengue virus type I (*Flaviviridae*) and vesicular stomatitis virus (*Paramyxoviridae*), and the non-enveloped virus human adenovirus-5 (*Adenoviridae*). Viruses were incubated with different concentrations of DIMPy-BODIPY and illuminated (400–700 nm, 65 ± 5 mW/cm^2^) as described or kept in the dark as a negative control. Virus infectivity after treatment was determined by plaque (VSV) or immunofocus assay (dengue, HAd-5) on Vero cells (Figure 2). In the absence of illumination, the infectivities of dengue and VSV were not affected at any of the concentrations of photosensitizer tested (Figure 2A). In contrast, after illumination, dengue virus (initially at 6.5 × 10^6^ FFU/mL) was inactivated below the level of detection at DIMPy-BODIPY concentrations of 1 μM and 0.1 μM, which represents a drop in infectivity of over 6 log units reduction in FFU/mL (*p* < 0.001). Even at the low concentration of 0.01 μM DIMPy-BODIPY, dengue was partially inactivated, with the infectivity showing a reduction of approximately 2 log units in FFU/mL (*p* < 0.001).

Pilot experiments demonstrated that VSV proved to be slightly more resistant to inactivation than dengue at 0.1 μM DIMPy-BODIPY, remaining almost fully infectious (unpublished data). Therefore, for VSV the concentrations of DIMPy-BODIPY were varied from 0.25 to 1 μM. As with dengue, the infectivity of VSV was not negatively affected at any of the DIMPy-BODIPY concentrations tested in the absence of illumination. After light treatment, however, VSV (initially at 10^7^ PFU/mL) was completely inactivated at 1 μM DIMPy-BODIPY, representing a drop in infectivity of over 6 log units reduction in PFU/mL (*p* < 0.001; Figure 2A). At 0.5 μM DIMPy-BODIPY, the viral infectivity was reduced nearly 5 log units in PFU/mL (*p* < 0.001), and was similarly reduced by nearly 4 log units in PFU/mL (*p* < 0.001) at the lowest effective concentration tested of 0.25 μM DIMPy-BODIPY.

In contrast to efficient photoinactivation of the enveloped viruses above, the non-enveloped human adenovirus-5 (initially at 6.5 × 10^5^ FFU/mL) proved more resistant to inactivation by DIMPy-BODIPY. At 1 μM, a 1 log unit reduction in FFU/mL (*p* < 0.025) was observed that increased to 2 log units (*p* < 0.001) at a photosensitizer concentration of 5 μM. A possible explanation to the greater resistance of adenovirus to photoinactivation is that the virus capsid in non-enveloped viruses, which in HAd-5 is comprised of at least nine different proteins (32), affords a greater level of protection to the virion from its surrounding environment than the lipid membrane and associated proteins of enveloped ones. Interestingly, the dark control samples themselves showed ~80% reduction in FFU/mL for 1–5 μM DIMPy-BODIPY, suggesting a non-photoinduced virus inactivation pathway may also be present, but this was not further explored. Finally, red blood cell hemolysis assays showed no statistically significant hemolytic property associated with DIMPy-BODIPY when tested to 10 μM concentration (Supplementary Table S1).

### 2.2. Antibacterial Photodynamic Inactivation Studies

**Figure 3:**
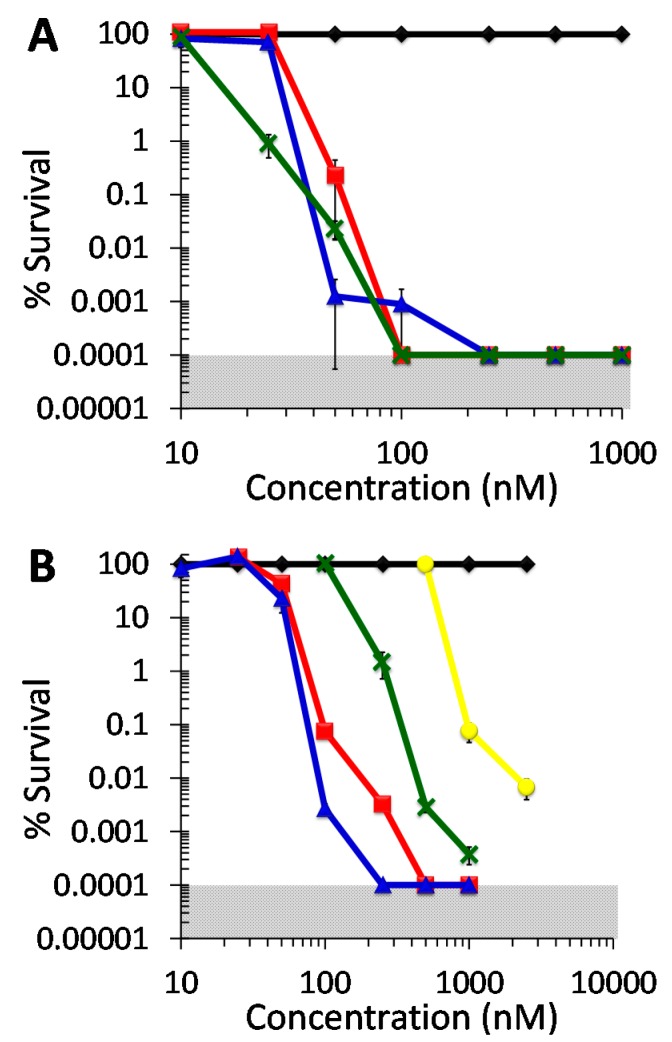
Photodynamic inactivation of bacteria as a function of DIMPy-BODIPY concentration. (**A**) Gram positive species. Displayed is the % survival of the dark control (♦) and the light treated samples for methicillin-susceptible *S. aureus* (MSSA) ATCC-2913 (■), methicillin-resistant *S. aureus* (MRSA) ATCC-44 (▲), and the vancomycin-resistant *Enterococcus faecium* (VRE) ATCC-2320 strain (x). (**B**) Gram negative species. Displayed is the % survival of the dark control (♦) and the light treated samples for *A. baumannii* ATCC-19606 (■), multidrug-resistant *A. baumannii* (MDRAB) ATCC-1605 (▲), *P. aeruginosa* ATCC-97 (x), and *K. pneumoniae* ATCC-2146 (●). For all bacteria, the illumination conditions were as follows: 30 min, 400–700 nm, 65 ± 5 mW/cm^2^ (total fluence of 118 J/cm^2^). As the plating technique employed to determine % survival did not allow for detection of survival rates of <0.0001%, data points below the detection limit were set to 0.0001% survival for graphing purposes. The shaded areas correspond to undetectable cell survival with the assay employed. In the cases where error bars cannot be visualized, the error bars themselves were smaller than the marker employed in the plot.

*In vitro* aPDI studies employing the photosensitizer DIMPy-BODIPY were performed in a concentration-dependent manner. The illumination time was fixed at 30 min for the bacterial studies as determined by a time-dependence study of the photobleaching of DIMPy-BODIPY (see Supplementary Data and Figure S1). All studies with bacteria employed a starting concentration of 1–4 × 10^8^ CFU/mL as determined by colony counting. For the three Gram-positive bacteria *S. aureus* ATCC-2913, methicillin-resistant *S. aureus* (MRSA) strain ATCC-44, and the vancomycin-resistant *E. faecium* (VRE) strain ATCC-2320, they were found to be highly susceptible to photodynamic inactivation with DIMPy-BODIPY regardless of their respective antibiotic resistance (Figure 3A). For example, at the concentration of 0.1 μM, DIMPy-BODIPY reduced bacterial survival by 5–6 log units (99.999+% viable cell eradication, *p* < 0.001) for all three after 30 min illumination (400–700 nm, 65 ± 5 mW/cm^2^). At concentrations above 0.25 μM under the same illumination conditions, no surviving bacteria were detected for any of the three bacteria. By comparison, the benchmark photosensitizers TMPyP (Supplementary Figure S3A) and methylene blue (Supplementary Figure S4A) showed no statistically significant cell inactivation at 0.1 μM photosensitizer concentration, and were only able to achieve a 5 log units reduction in bacteria when 5- to 10-fold higher concentrations of the photosensitizers were employed (typically 1 μM or higher). See Supplementary Figure S5 for a comparison of their electronic absorption spectra and that of DIMPy-BODIPY. Decreasing the DIMPy-BODIPY concentration to 0.05 μM led to a partial attenuation of the inactivation efficacy for both *S. aureus* ATCC-2913 (2.5 log units reduction in CFU/mL, *p* < 0.001) and *E. faecium* ATCC-2320 (3.5 log units reduction, *p* < 0.001), whereas there was no statistical difference observed for the MRSA strain ATCC-44 between 0.05 and 0.1 μM concentrations (both ~5 log units reduction). When the concentration of the photosensitizer was further decreased to 0.025 μM, each of the *S. aureus* strains ceased to be inactivated, while the *E. faecium* strain remarkably still exhibited 99% inactivation (2 log units, *p* < 0.001). No statistically significant inactivation was seen at a DIMPy-BODIPY concentration of 0.01 μM for any of the three aforementioned bacteria.

**Figure 4:**
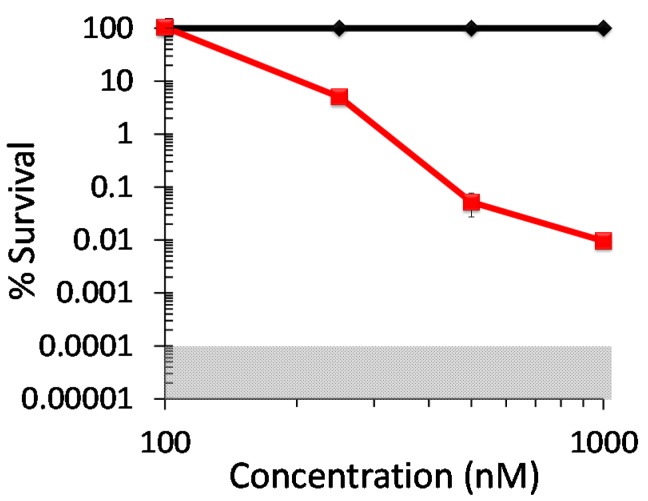
Photodynamic inactivation of *Mycobacterium smegmatis* mc^2^155 ATCC-700084 as a function of DIMPy-BODIPY concentration. Displayed is the % survival of the dark control (♦) and the light treated samples (■). Illumination and assay conditions were as in Figure 3.

The taxonomically Gram-positive bacterium *M. smegmatis* strain ATCC-700084, shown separately in Figure 4, exhibited ~4 log units inactivation at 1 μM (*p* < 0.001), ~3.3 log units at 0.5 μM (*p* < 0.001), and 95% inactivation (~2 log units, *p* < 0.001) at 0.25 μM. No statistically significant inactivation of this *M. smegmatis* strain was observed at DIMPy-BODIPY concentrations below 0.1 μM. By comparison, methylene blue is a much poorer photosensitizer under identical illumination conditions, having previously been shown to photoinactivate *M. smegmatis* by 4 log units at only a very high concentration of 75 μM, by 2 log units at 7.5 μM, and showed no inactivation at 0.75 μM [13]. However, under the same conditions, TMPyP has been shown to be highly efficient in its photoinactivation of *M. smegmatis*, achieving detection limit inactivation (6+ log units reduction) at concentrations as low as 0.15 μM [13].

While the Gram-positive species were fairly consistent with respect to their photoinactivation when compared to one another, the Gram-negative ones (drug-susceptible *A. baumannii* strain ATCC-19606, the multi-drug resistant *A. baumannii* (MDRAB) strain ATCC-1605, *P. aeruginosa* strain ATCC-97 and *K. pneumoniae* strain ATCC-2146) had a wider range of susceptibilities to BODIPY-mediated aPDI (Figure 3B). When the efficacy of DIMPy-BODIPY at a concentration of 0.5 μM was examined against the two *A. baumannii* strains, no CFUs were detected, and survival rates were therefore below the detection limit of <0.0001%, corresponding to an impressive 6 log units reduction in viable cells (*p* < 0.001). At 0.1 μM, DIMPy-BODIPY was still able to achieve a noteworthy 3 and 4.5 log units reduction in CFU for *A. baumannii* ATCC-19606 and MDRAB ATCC-1605, respectively, whereas 0.05 μM was the lowest PS concentration for which aPDI was statistically significant (57% and 77% cell eradication for *A. baumannii* ATCC-19606 and MDRAB ATCC-1605, respectively, *p* < 0.02). No statistically significant inactivation of either strain was seen at a DIMPy-BODIPY concentration of 0.025 μM or lower. Thus, with the exceptions at the extremes of the concentrations studied (above 0.5 μM or below 0.025 μM), the MDRAB ATCC-1605 strain was statistically more susceptible (*p* = ~0.02 or lower), albeit only slightly, to photodynamic inactivation by DIMPy-BODIPY when compared with *A. baumannii* ATCC-19606. By comparison, the commercial photosensitizers were poorer performers: TMPyP (Supplementary Figure S3B) required a 5-fold higher concentration (2.5 μM) to achieve 5 log units of inactivation, and methylene blue (Supplementary Figure S4B) required a 2-fold higher concentration (1.0 μM), with both exhibiting no statistically significant photoinactivation at or below 0.25 μM.

The *P. aeruginosa* ATCC-97 strain was also found to be inactivated by DIMPy-BODIPY at sub-μM to low μM concentrations (Figure 3B): ~5.5 log units inactivation was observed (*p* < 0.001) at a photosensitizer concentration of 1 μM. Lowering the concentration to the sub μM regime decreased the inactivation efficacy; however, it was still possible to achieve a notable ~4.5 log units reduction in CFU/mL at 0.5 μM (*p* < 0.001), and 98% inactivation (~2 log units, *p* < 0.001) at 0.25 μM. No statistically significant inactivation of this *P. aeruginosa* strain was observed at DIMPy-BODIPY concentrations below 0.1 μM. As a benchmark, methylene blue was able to match the highest observed level of photoinactivation (~5.5 log units; Supplementary Figure S4B), but required a 5-fold higher photosensitizer concentration (5 μM) to do so, whereas TMPyP (Supplementary Figure S3B) was only able to achieve 3 log units reduction in viable cells at that same concentration.

In comparison to the other bacterial species investigated, the multi-drug resistant NDM-1-producing *K. pneumoniae* clinical isolate ATCC-2146 explored here was the least susceptible to photodynamic inactivation by DIMPy-BODIPY, with no inactivation observed at 0.5 μM (Figure 3B). At 1 μM, a concentration that achieved a detection-limit level of inactivation for nearly all other bacteria examined, a 99.9% (3 log units) reduction in CFU/mL was obtained for this *K. pneumoniae* strain (*p* < 0.001). In an attempt to see if near detection limit inactivation was possible, the photosensitizer concentration was increased to 2.5 μM, which showed ~4 log units of inactivation (>99.99%, *p* < 0.001). By comparison, TMPyP was slightly more effective than DIMPy-BODIPY, reaching ~5 log units reduction at 2.5 μM (Supplementary Figure S3B), whereas methylene blue was a far poorer photosensitizer, exhibiting no statistically significant photoinactivation at that concentration (Supplementary Figure S4B).

The two main pathways by which aPDI inactivates microbes include: The Type I mechanism (redox), where the photosensitizer in its triplet state undergoes electron transfer reactions with suitable substrates such as cellular biomolecules, solvent molecules or dioxygen, forming cytotoxic free radicals and superoxide; and the Type II mechanism (energy transfer), where energy transfer from the excited triplet state of the photosensitizer to ground state triplet molecular oxygen leads to the formation of highly reactive singlet oxygen [36]. In order to probe whether DIMPy-BODIPY mediates aPDI through, in part, a Type II mechanism for photosensitization, the singlet oxygen quantum yield was measured in methanol by direct observation of the singlet oxygen phosphorescence, and was found to be 11%. This is in comparison with the Φ = 19% value previously reported in isopropanol [26]. Moreover, the known singlet oxygen quencher sodium azide was employed in cell survival studies [13]. When the aPDI assay was repeated for *A. baumannii* in the presence of 0.2–20 mM NaN_3_, a statistically significant increase in cell survival was observed for DIMPy-BODIPY (Supplementary Figure S2, Supplementary Data), suggesting that aPDI of *A. baumannii* (ATCC-19606) is mediated in part by singlet oxygen production. Cell survival was increased at the sodium azide concentration of 0.2 mM, and the greatest increase was noted at 20 mM: Specifically, only 3 log units of inactivation were observed in the presence of the singlet oxygen quencher *vs.* 6 log units in its absence. Above 20 mM, control experiments showed the azide inhibited growth of the bacterium (unpublished data), and for this reason higher concentrations of azide were not pursued due to its toxicity as a metabolic inhibitor. Although the data strongly suggest a Type II mechanism of aPDI by DIMPy-BODIPY, contributions from a Type I pathway cannot be ruled out.

Despite knowing that *in situ* formed singlet oxygen is the likely microbiocidal reagent, we are unable to determine if strain-specific variances or species-specific differences are responsible for the different aPDI inactivation efficiencies observed for the Gram-negative bacteria investigated in this study. Given that the photoinactivation of the Gram-positive bacteria was fairly consistent, one obvious factor may be different compositions and properties associated with the bacterial outer membrane of the Gram-negative bacteria. This reasoning could explain, for example, why *M. smegmatis* was more difficult to photoinactivate when compared to *A. baumannii* or *P. aeruginosa* given that its cell wall is thicker than in many other bacteria, as well as more hydrophobic and waxy due to it being rich in mycolic acids/mycolates [37]. Different liposaccharide compositions could also result in variations of the number/density of negative charges present in the outer membrane, which in turn would affect cationic photosensitizer binding, whereas differences in the porins may also affect photosensitizer uptake. Finally, variations in the number and efficiency of efflux pumps, particularly important for antibiotic resistance in *K. pneumoniae* [38], may also be a factor in expelling photosensitizers that may be uptaken by the bacteria.

### 2.3. Antifungal Photodynamic Inactivation Studies

**Figure 5:**
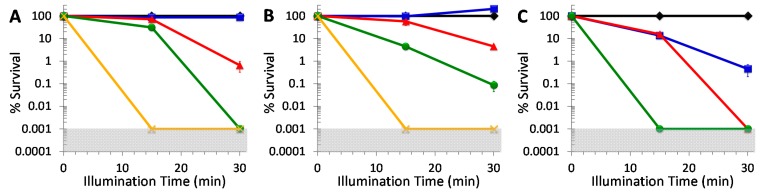
Photodynamic inactivation of (**A**) *Candida albicans* ATCC-90028, (**B**) *Candida glabrata* ATCC-15545, and (**C**) *Cryptococcus neoformans* ATCC-64538 as a function of DIMPy-BODIPY concentration and illumination time. Displayed is the % survival of the dark control (♦) and the light treated samples at 0.1 μM (■), 0.5 μM (▲), 1 μM (●), and 5 μM (X). Illumination conditions were as follows: 400–700 nm, 65 ± 5 mW/cm^2^, and either 15 or 30 min (total fluences of 59 and 118 J/cm^2^, respectively). As the plating technique employed to determine % survival did not allow for detection of survival rates of <0.001%, data points below the detection limit were set to 0.001% survival for graphing purposes. The shaded areas correspond to undetectable cell survival with the assay employed. In the cases where error bars cannot be visualized, the error bars themselves were smaller than the marker employed in the plot.

Two opportunistic *Candida* pathogens, the *C. albicans* strain ATCC-90028 (Figure 5A) and the *C. glabrata* strain ATCC-15545 (Figure 5B), proved to be similar in their susceptibilities to photoinactivation with DIMPy-BODIPY. Each strain, initially at ~10^7^ CFU/mL, showed detection limit inactivation (5 log units CFU reduction) with 5 μM DIMPy-BODIPY and either 15 or 30 min of illumination (*p* < 0.001). 

For studies performed at 1 μM concentration, the *C. albicans* strain exhibited 69% cell inactivation after 15 min of illumination (*p* < 0.001), and proved highly susceptible upon an increase in illumination time, with a full 5 log units of inactivation to the detection limit with 30 min illumination. The *C. glabrata* strain was 95% (*p* < 0.001) inactivated after 15 min illumination, though it only exhibited a slight increase to 3 log units inactivation (*p* < 0.001) at the longer 30 min illumination time. The *C. albicans* strain (99.5%, ~2 log units, *p* < 0.001) was also inactivated more efficiently than the *C. glabrata* one (95%, ~1 log unit, *p* < 0.001) at 30 min of illumination and 0.5 μM DIMPy-BODIPY concentration. No inactivation of the two *Candida* strains was observed for either 15 or 30 min illumination at a photosensitizer concentration of 0.1 μM. Overall, these results with DIMPy-BODIPY compared favorably against the commercial photosensitizer methylene blue (for *C. albicans*, no inactivation was observed with 1 μM methylene blue, and ~4.5 log units at 5 μM PS concentration (Supplementary Figure S6A); for *C. glabrata*, ~3 log units reduction at 5 μM (Supplementary Figure S7A), and were comparable to the detection limit inactivation observed for TMPyP at 5 μM using either 15 or 30 min of illumination for *C. albicans* (Supplementary Figure S6B) and *C. glabrata* (Supplementary Figure S7B).

The yeast pathogen *Cryptococcus neoformans* ATCC-64538, whose species gives rise to an estimated one million cases of meningitis resulting in 625,000 deaths worldwide each year [39], proved to be more susceptible to photoinactivation with DIMPy-BODIPY than either of the *Candida* species (Figure 5C). Starting with an initial concentration of ~10^7^ CFU/mL, detection limit inactivation (5 log units CFU reduction) was achieved at a photosensitizer concentration of 1 μM with 15 min illumination, and at 0.5 μM with the longer 30 min illumination (*p* < 0.001). Remarkably, even at the lowest concentration examined of 0.1 μM DIMPy-BODIPY, a noteworthy 99.5% (2+ log units, *p* < 0.001) reduction in CFU was observed for this *C. neoformans* strain for the 30 min illumination time. Again, these results for DIMPy-BODIPY compare favorably against the benchmark photosensitizers methylene blue (Supplementary Figure S8A) and TMPyP (Supplementary Figure S8B), both of which were able to reach detection limit inactivation, but required a 5-fold higher concentration of 5 μM to do so when compared with DIMPy-BODIPY.

The difference in photoinactivation efficacy between the three different yeast strains may be attributed to differences in their extracellular structures. *C. neoformans* is encapsulated and known to produce melanin, while Candida species do not. The capsule contains highly negative charged polysaccharides found immediately outside the cell wall [40,41,42], namely glucuronoxylomannan (GXM), galactoxylomannan (GalXM) and mannoprotein, where GXM makes up approximately 90% of the capsule composition and ranges from 1 to 50 μm in thickness [43]. Both the polysaccharide and melanin production result in a strong negative charge on the cell surface. The high photodynamic inactivation efficacy of the cationic DIMPy-BODIPY towards *C. neoformans* may be explained by a stronger electrostatic interaction between the cell surface and the photosensitizer than with the Candida species.

## 3. Experimental Section

### 3.1. Materials

Buffer salts and methylene blue were purchased from Fisher Scientific (Pittsburgh, PA, USA) Nutrient Broth #234000 was obtained from BD Difco (Franklin Lakes, NJ, USA), LB broth Miller from EMD Chemicals (Billerica, MA, USA), tetramethylpyridylporphyrin tetratosylate from Frontier Scientific, and Tryptic Soy Broth from Teknova (Hollister, CA, USA). Polylysine solution was purchased from Sigma Aldrich (St. Louis, MO, USA). Unless otherwise specified, all other chemicals were obtained from commercial sources in the highest purity available. Ultrapure water used for all media and buffers was provided by an Easypure II system (Barnstead, Dubuque, IA, USA). UV-visible absorption measurements were performed on a Cary 50 Bio instrument (Varian, Santa Clara, CA, USA) or a Genesys 10 UV scanning spectrophotometer from Thermo Electron Corp. (Waltham, MA, USA) for single wavelength measurements. The photosensitizer 2,6-diiodo-1,3,5,7-tetramethyl-8-(*N*-methyl-4-pyridyl)-4,4′-difluoroboradiazaindacene [DIMPy-BODIPY; log *p* = −1.96; λ_max_ (H_2_O) = 509 nm (75.9 mM^−1^·cm^−1^)] was synthesized per the published protocol [26,30,44].

### 3.2. Cell Culture

All bacteria were grown in 5 mL cultures incubated at 37 °C on an orbital shaker under the following growth conditions: *Acinetobacter baumannii* (ATCC-19606) was grown in Miller-LB media without antibiotics; the multi-drug resistant strain of *Acinetobacter baumannii* (ATCC-1605) was grown in Miller-LB media with 5 μg/mL tetracycline; methicillin-susceptible *Staphylococcus aureus* 2913 was grown in tryptic soy broth media without antibiotics; methicillin-resistant *Staphylococcus aureus* (ATCC-44) was grown in tryptic soy broth media with 5 μg/mL tetracycline; *Pseudomonas aeruginosa* (ATCC-97) was grown in BD Difco Nutrient Broth #234000 with 5 μg/mL tetracycline; *Mycobacterium smegmatis* mc^2^155 was grown in BD Difco 7H9 media with ADS and 100 μg/mL cycloheximide; *Klebsiella pneumoniae* (ATCC-2146) was grown in BD Difco Nutrient Broth #234000 with 100 μg/mL ampicillin. The vancomycin-resistant strain of *Enterococcus faecium* (ATCC-2320) was grown in BD Difco Bacto Brain Heart Infusion 237,500 with 100 μg/mL ampicillin. Each bacterium was grown to a concentration of 1–4 × 10^8^ CFU/mL (determined spectrophotometrically from growth curves using a Genesys 10 UV scanning spectrophotometer) prior to being pelleted by centrifugation (10 min, ~3700 *g*). Once pelleted, the supernatant was decanted and the cells were resuspended in 5 mL of PBS (170 mM NaCl, 3.4 mM KCl, 10.0 mM Na_2_HPO_4_, 1.8 mM KH_2_PO_4_, pH 7.2) containing 0.05% Tween-80 (to prevent agglomeration of *M. smegmatis* [45]) and diluted to ~10^8^ CFU·mL^−1^ (determined spectrophotometrically). *Candida albicans* (ATCC-90028) and *Candida glabrata* (ATCC-15545) were grown aerobically overnight in yeast extract-peptone-dextrose (YPD) broth at 37 °C. *Cryptococcus neoformans* (ATCC-64538) was grown aerobically in Sabouraud dextrose broth at 30 °C for 48 h. Cells were harvested by centrifugation (10 min, ~3700 *g*) and washed twice with PBS. The cells were resuspended in PBS and diluted to ~10^7^ CFU·mL^−1^ (determined spectrophotometrically).

### 3.3. Viral Propagation

Vesicular stomatitis virus (VSV) NJ strain was propagated on Vero cells and titered by plaque assay on Vero cells. Dengue 1 virus was propagated on C6/36 mosquito cells and titered on Vero cells by immunofocus assay. Human adenovirus-5 (HAd-5) was propagated on the human lung carcinoma cell line A549 and titered on the same cells.

### 3.4. Photodynamic Inactivation Assay

All photosensitization experiments were performed using a non-coherent light source, PDT light model LC122 (LumaCare, Newport Beach, CA, USA), equipped with a LUM V fiber optic probe (400–700 nm band pass filter, average transmittance T_avg_ ~95% ± 3%) and an OSRAM Xenophot lamp model 64653 HLX (24 V, 250 W). The fluence rate was measured with an Orion power meter (Orphir Optronics Ltd, Jerusalem, Israel). All experiments were conducted in triplicate at a minimum, and statistical significance was assessed via a two-tailed, unpaired Student’s *t*-test. Sterile stock solutions of DIMPy-BODIPY were prepared in filter sterilized ultrapure water.

#### 3.4.1. Bacteria and Yeast

Five mL cultures were incubated with DIMPy-BODIPY (0.01–2.5 μM final concentration as indicated in Figure 3 and Figure 4 for bacteria, and 0.01–5 μM final concentration for yeast) on an orbital shaker in the dark for 5 (bacteria) or 15 (yeast) min. After incubation, three 1 mL aliquots of the cell suspension were transferred to a sterile 24-well plate (BD Falcon, flat bottom) and illuminated with visible light (400–700 nm) with a fluence rate of 65 ± 5 mW/cm^2^ for a variable period of time (5–60 min) while magnetically stirred. The remaining aliquots of cell culture were kept in the absence of light as the dark control. Studies were repeated in the absence of the photosensitizer as a no compound light control (Supplementary Table S2). After illumination, each well was 1:10 serially diluted five times. 10 μL from the undiluted well and from each dilution, as well as from the dark control, were plated and incubated in the dark at 37 °C. Each bacterium was grown on gridded six column square agar plates made with their respective growth media without antibiotics, with the exception of *M. smegmatis*, which was plated on BD Difco 7H10-ADS containing 100 μg/mL cycloheximide. The survival rate was determined from the ratio of CFU/mL of the illuminated solution *vs.* that of the dark control. The minimum detection limit was 100 CFU/mL (based on 10 μL plated from the 1 mL undiluted well). Variations in the concentration of the starter culture (1–4 × 10^8^ CFU/mL for bacteria, and 1–4 × 10^7^ CFU/mL for yeast) resulted in a variation of the detection limit spanning the region of 0.001%–0.0001% survival for bacteria, and 0.01%–0.001% for yeast, respectively. Samples with PS present but kept in the dark (dark control) and illuminated samples without PS (light control) served as controls.

#### 3.4.2. Vesicular Stomatitis Virus

VSV (10^7^ plaque forming units, PFU) of were incubated with DIMPy-BODIPY (0.01–1 μM final concentration) for 5 min in the dark prior to 30 min under visible light illumination (400–700 nm; 65 ± 5 mW/cm^2^) in a total volume of 100 mL of MEM supplemented with 10 mM HEPES, 1% FBS and antibiotics. Control experiments were similarly performed in the dark. Aliquots of virus samples were subsequently titered on Vero cells, and the virus concentration was determined by plaque assay (detection limit of 40 PFU/mL). Specifically, samples were set up in biological duplicates, viruses were titered by serial 10-fold dilution on Vero cells in 24-well plates at 37 °C. Plaques were detected by crystal violet staining 48 h after infection. Where virus was detectable, the plaques at dilutions where wells contained between 10 to 20 plaques were counted for titer determination. At concentrations of DIMPy-BODIPY where the virus sample was largely inactivated it was necessary to use wells containing fewer plaques. The limit of detection was 40 PFU/mL.

#### 3.4.3. Dengue-1 Virus

The virus (6.5 × 10^6^ focus forming units, FFU) was incubated with DIMPy-BODIPY (0.25–1 μM final concentration) for 5 min in the dark prior to 30 min under visible light illumination (400–700 nm; 65 ± 5 mW/cm^2^) in a total volume of 100 mL of MEM supplemented with 10 mM HEPES, 1% FBS and antibiotics. Control experiments were similarly performed in the dark. Samples set up in biological triplicate were used. Virus was titered by serial 10-fold dilution on A549 cells in 24-well plates at 37 °C for 72 h. Immunofoci were detected with an antibody to the E1A protein and a secondary anti-mouse antibody conjugated to horseradish peroxidase. Where virus was detectable, immunofoci at dilutions where wells contained between 10 to 20 foci were counted for titer determination. At concentrations of DIMPy-BODIPY where the virus sample was largely inactivated it was necessary to use wells containing fewer foci. The detection limit was 50 FFU/mL.

#### 3.4.4. Human Adenovirus-5

The virus (6.5 × 10^5^ focus forming units, FFU) was incubated with DIMPy-BODIPY (0.25–1 μM final concentration) for 5 min in the dark prior to 30 min under visible light illumination (400–700 nm; 65 ± 5 mW/cm^2^) in a total volume of 100 mL of MEM supplemented with 10 mM HEPES, 1% FBS and antibiotics. Control experiments were similarly performed in the dark. Samples set up in biological triplicate were used. Virus was titered by serial 10-fold dilution on A549 cells in 24-well plates at 37 °C for 72 h. Immunofoci were detected with an antibody to the E1A protein and a secondary anti-mouse antibody conjugated to horseradish peroxidase. Where virus was detectable, immunofoci at dilutions where wells contained between 10 to 20 foci were counted for titer determination. At concentrations of DIMPy-BODIPY where the virus sample was largely inactivated it was necessary to use wells containing fewer plaques. The detection limit was 66 FFU/mL.

### 3.5. Red Blood Cell Hemolysis Assay

Hemolysis assays employing mechanically defibrinated sheep blood were performed following literature protocol [46], using the following concentrations of DIMPy-BODIPY: 0.05, 0.1, 0.5, 1, 5 and 10 μM. A compound-free control was performed to verify the background, and a 2% (*v*/*v*) Triton X in 0.5 mL PBS was used as the 100% lysis positive control.

### 3.6. Singlet Oxygen Quantum Yield Determination

Singlet oxygen formation quantum yield measurements were performed in aerated solutions of methanol in a 1 cm^2^ quartz cell at ambient temperature with an Edinburgh fluorimeter (FS920) and near-IR photomultiplier tube. The resulting value for the singlet oxygen phosphorescence of DIMPy-BODIPY was compared to Rose Bengal (Φ = 0.80 [47]) in air-saturated methanol with a 514.5 nm excitation wavelength at a power of 250 mW. The quantum yield was calculated as previously described [48].

## 4. Conclusions

We have now further investigated the ability of BODIPY-based photosensitizers to photoinactivate bacteria, fungi, and viruses using visible light (400–700 nm). Although we cannot rule out strain-specific results given the limited numbers of pathogens explored herein, the results obtained demonstrate that DIMPy-BODIPY is able to mediate the photodynamic inactivation of clinically-relevant microbes, including Gram-positive, Gram-negative, and drug-resistant bacteria, as well as pathogenic yeast and model viruses, at nanomolar concentrations and short illumination times. With minor exceptions, DIMPy-BODIPY performed more efficiently over the entire range of microbes studied when compared to the benchmark standards of methylene blue or TMPyP, thus highlighting the utility of the BODIPY-class of photosensitizers for a broad spectrum of potential aPDI application, particularly when one considers the possible synthetic modifications to this modular scaffold. In addition to their potential for *in vivo* antimicrobial photodynamic therapy, BODIPY-based chromophores may represent an additional class of photosensitizers for incorporation in photomicrobiocidal materials for the elimination of pathogens from surfaces prior to their transmission to hosts [49], and future studies are planned to demonstrate the potential of this new class of photosensitizer for application as a more broadly applicable anti-infective agent.

## Supplementary Materials

Illumination time dependence study (Supplementary Figure S1), sodium azide singlet oxygen quenching study (Supplementary Figure S2), red blood cell hemolysis assay (Supplementary Table S1), light control data (Supplementary Table S2), PDI of bacteria by TMPyP (Supplementary Figure S3) and methylene blue (Supplementary Figure S4), electronic absorption spectra of DIMPy-BODIPY, methylene blue, and TMPyP (Supplementary Figure S5), and PDI of yeast by TMPyP and methylene blue (Supplementary Figures S6–S8) are also provided.

Supplementary materials can be accessed at: http://www.mdpi.com/1420-3049/20/06/10604/s1.
