# Supplementary material for: Antiviral, Antifungal and Antibacterial Activities of a BODIPY-Based Photosensitizer
Source: Molecules. 2015 Jun 8;20(6):10604–21. doi: 10.3390/molecules200610604 (PMC6272413; doi:10.3390/molecules200610604)
Supplement: Supplementary file 1 [file molecules-20-10604-s001.pdf]

Supporting Information

Photobleaching/Illumination Time Dependence Study

The time-dependence of illumination on the photobleaching of DIMPy-BODIPY as determined by the resultant loss in aPDT efficacy was undertaken using drug-susceptible *A. baumannii*. The photosensitizer concentration was held constant at 100 nM while the illumination time was varied between 5 and 60 min. As seen in Figure S1, an illumination period of 5 min resulted in no statistically significant inactivation. When the illumination time was extended to 15 min, ~60% of the bacteria were inactivated, and when extended to 30 min greater than 90% were inactivated. Extending the illumination time to 45 or 60 min however, showed no statistically significant inactivation beyond that of the 30 min illumination.

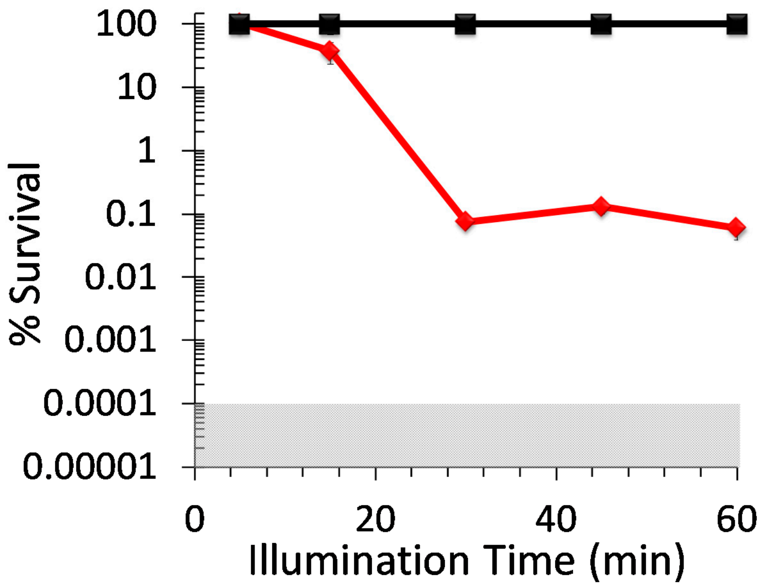

Figure S1. DIMPy-BODIPY-mediated photodynamic inactivation of *A. baumannii* as a function of the illumination time. The studies were performed in the presence of 100 nM of the photosensitizer. Displayed is the % survival of the dark control (♦) and the light treated samples (■). Illumination conditions were as follows: 5–60 min, 400–700 nm, 65 ± 5 mW/cm^2 (total fluences of 20–236 J/cm^2). As the plating technique employed to determine % survival did not allow for detection of survival rates of <0.0001%, data points below the detection limit were set to 0.0001% survival for graphing purposes. The shaded areas correspond to undetectable cell survival with the assay employed. In the cases where error bars cannot be visualized, the error bars themselves were smaller than the marker employed in the plot.

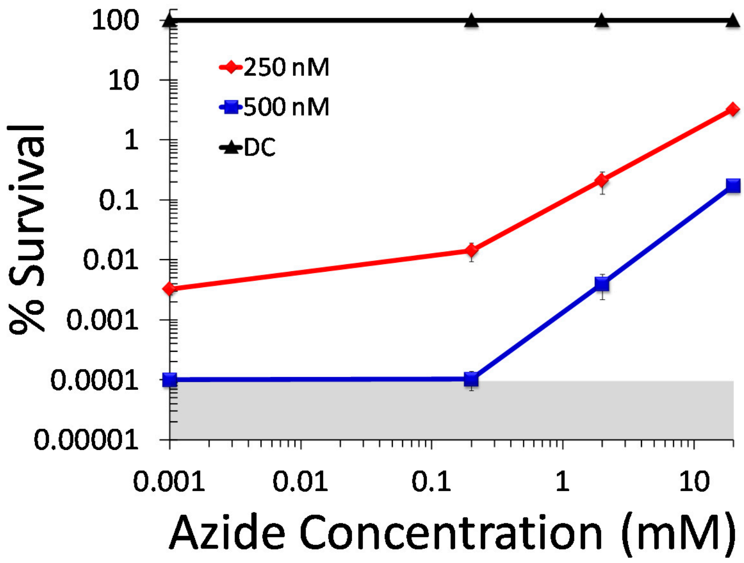

Figure S2. DIMPy-BODIPY-mediated photodynamic inactivation of *A. baumannii* as a function of the concentration of the singlet oxygen quencher sodium azide (0.001–20 mM). The studies were performed at 0 nM (as dark control, ▲), 250 nM (♦), and 500 nM (■) concentrations of the photosensitizer. Illumination conditions were as follows: 30 min, 400–700 nm, 65 ± 5 mW/cm^2 (total fluence of 118 J/cm^2). As the plating technique employed to determine % survival did not allow for detection of survival rates of <0.0001%, data points below the detection limit were set to 0.0001% survival for graphing purposes. The shaded areas correspond to undetectable cell survival with the assay employed. In the cases where error bars cannot be visualized, the error bars themselves were smaller than the marker employed in the plot.

Table S1. Red blood cell hemolysis assay with DIMPy-BODIPY.

[DIMPy-BODIPY] (µM)	Raw Abs^{5400}	Corrected Abs^{5400} [a]
0 µM	0.0084	n/a [b]
0.05 µM	0.0087	0.0086
0.1 µM	0.0075	0.0061
0.5 µM	0.0082	0.0070
1 µM	0.0122	0.0098
5 µM	0.0243	0.0122
10 µM	0.0299	0.0058
100% lysed	0.9682	n/a [b]

[a] corrected = raw Abs^{5400} – Abs^{5400} from DIMPy-BODIPY itself in PBS buffer; [b] n/a = not applicable as no DIMPy-BODIPY was present in this measurement.

Table S2. Cell or virus survival under illumination-only conditions (light control).

Bacterium	Survival (% ± S.D.)
Staphylococcus aureus	85 ± 21
**MRSA**	93 ± 12
**VRE**	106 ± 24
Acinetobacter baumannii	123 ± 18
**MDRAB**	76 ± 28
Pseudomonas aeruginosa	89 ± 9
Klebsiella pneumoniae	79 ± 8

Virus	
**dengue**	66 ± 17
**HAd-1**	107 ± 23
**VSV**	103 ± 22

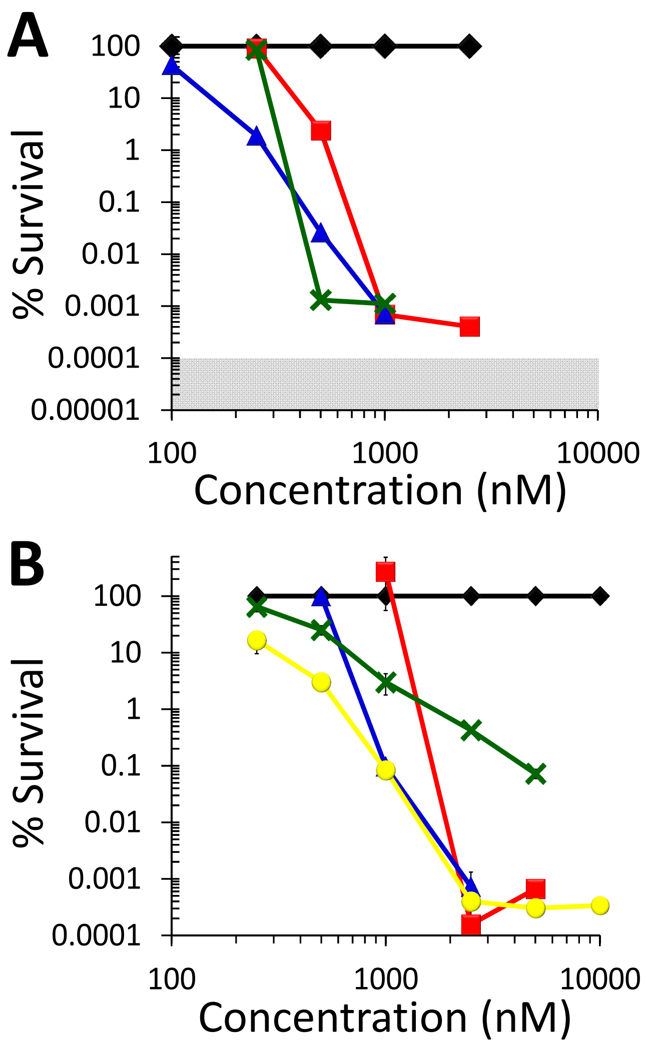

Figure S3. Photodynamic inactivation of bacteria as a function of TMPyP concentration. (**A**) Gram positive species. Displayed is the % survival of the dark control (♦) and the light treated samples for methicillin-susceptible *S. aureus* (MSSA) ATCC-2913 (■), methicillin-resistant *S. aureus* (MRSA) ATCC-44 (▲), and the vancomycin-resistant *Enterococcus faecium* (VRE) ATCC-2320 strain (**x**). (**B**) Gram negative species. Displayed is the % survival of the dark control (♦) and the light treated samples for *A. baumannii* ATCC-19606 (■), multidrug-resistant *A. baumannii* (MDRAB) ATCC-1605 (▲), *P. aeruginosa* ATCC-97 (**x**), and *K. pneumoniae* ATCC-2146 (●). For all bacteria, the illumination conditions were as follows: 30 min, 400–700 nm, 65 ± 5 mW/cm^2 (total fluence of 118 J/cm^2). As the plating technique employed to determine % survival did not allow for detection of survival rates of <0.0001%, data points below the detection limit were set to 0.0001% survival for graphing purposes. The shaded areas correspond to undetectable cell survival with the assay employed. In the cases where error bars cannot be visualized, the error bars themselves were smaller than the marker employed in the plot.

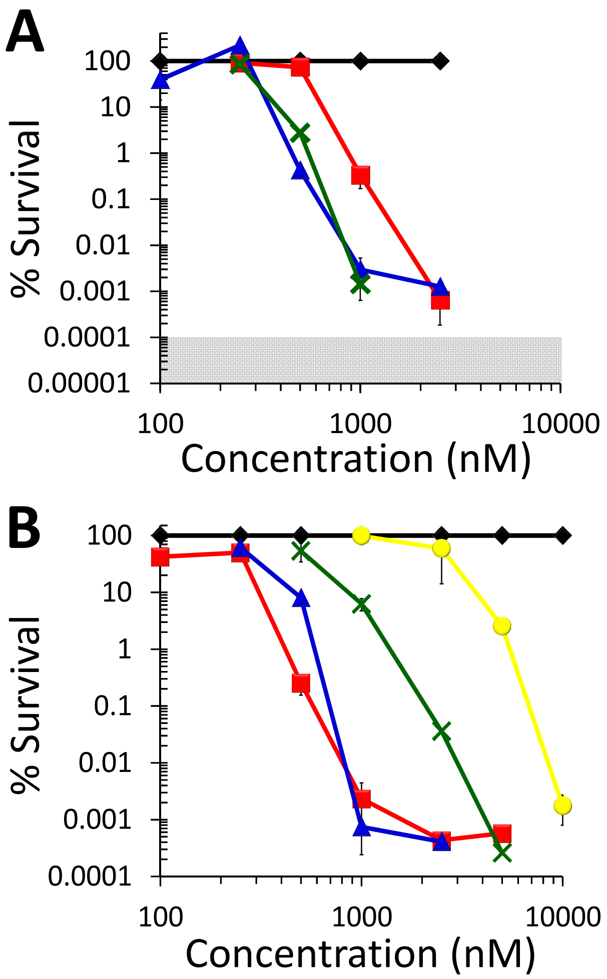

Figure S4. Photodynamic inactivation of bacteria as a function of methylene blue concentration. (**A**) Gram positive species. Displayed is the % survival of the dark control (♦) and the light treated samples for methicillin-susceptible *S. aureus* (MSSA) ATCC-2913 (■), methicillin-resistant *S. aureus* (MRSA) ATCC-44 (▲), and the vancomycin-resistant *Enterococcus faecium* (VRE) ATCC-2320 strain (**x**). (**B**) Gram negative species. Displayed is the % survival of the dark control (♦) and the light treated samples for *A. baumannii* ATCC-19606 (■), multidrug-resistant *A. baumannii* (MDRAB) ATCC-1605 (▲), *P. aeruginosa* ATCC-97 (**x**), and *K. pneumoniae* ATCC-2146 (●). For all bacteria, the illumination conditions were as follows: 30 min, 400–700 nm, 65 ± 5 mW/cm^2 (total fluence of 118 J/cm^2). As the plating technique employed to determine % survival did not allow for detection of survival rates of <0.0001%, data points below the detection limit were set to 0.0001% survival for graphing purposes. The shaded areas correspond to undetectable cell survival with the assay employed. In the cases where error bars cannot be visualized, the error bars themselves were smaller than the marker employed in the plot.

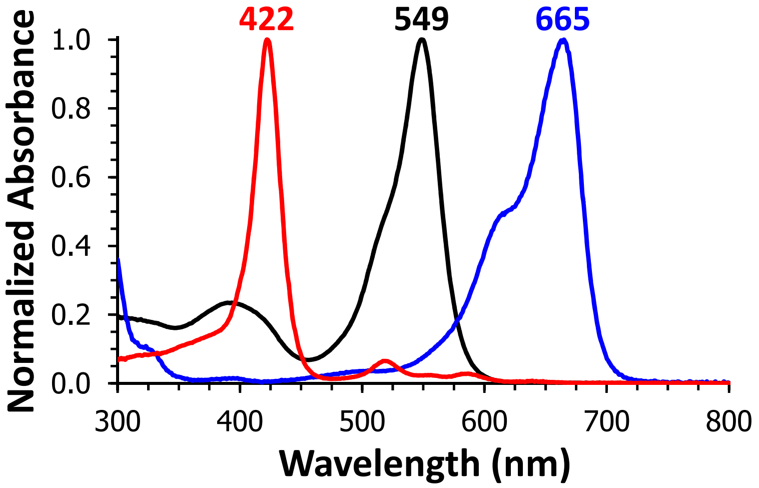

Figure S5. Electronic absorption spectra (normalized) of DIMPy-BODIPY (black; ε_{549nm} = 97,500 $M^{-1}\cdot cm^{-1}$), methylene blue (blue; ε_{664nm} = 81,000 $M^{-1}\cdot cm^{-1}$ [1]), and TMPyP (red; ε_{424nm} = 226,000 $M^{-1}\cdot cm^{-1}$ [2]) in water. The spectrum of the LumaCare LC-122 illumination source [400–700 nm, average transmittance (T_{avg}) ~95% ± 3%] overlaps with the main absorption band for each photosensitizer as follows: DIMPy-BODIPY, >99%; methylene blue, ~98%; TMPyP, ~87%. The molar absorption coefficient measured here in water is in good agreement with that previously reported for DIMPy-BODIPY in acetone of ε_{546nm} = 110,000 $M^{-1}\cdot cm^{-1}$ [3].

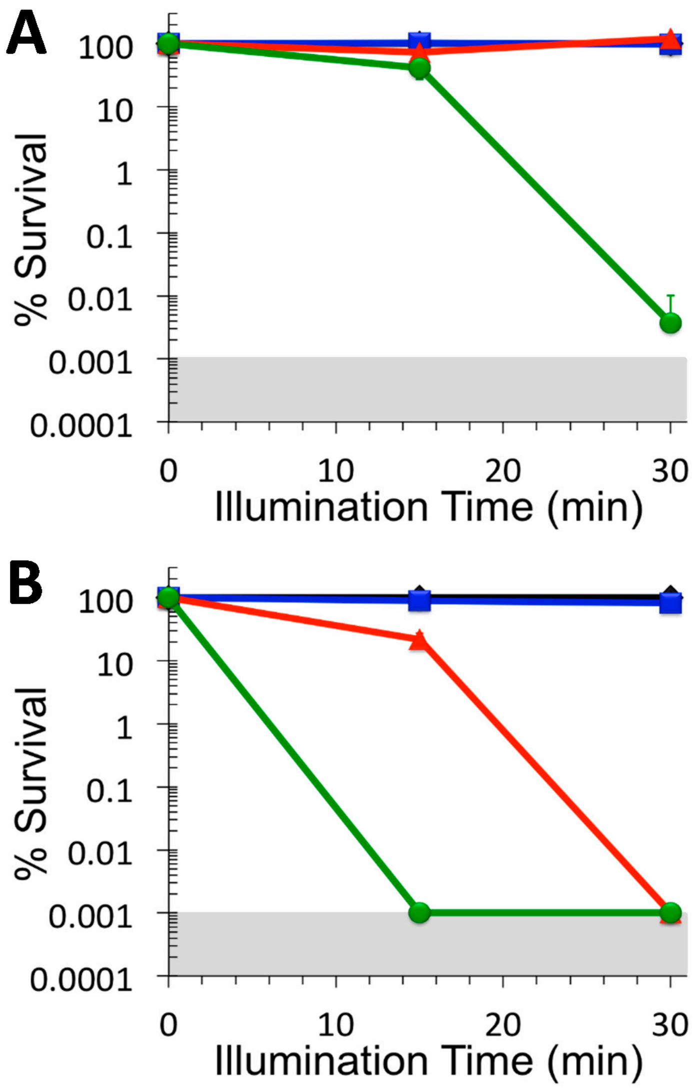

Figure S6. Photodynamic inactivation of *Candida albicans* ATCC-90028 as a function of photosensitizer concentration and illumination time for A) methylene blue and B) TMPyP. Displayed is the % survival of the dark control (♦) and the light treated samples at 0.1 μM (■), 1 μM (▲), and 5 μM (●). Illumination conditions were as follows: 400–700 nm, 65 ± 5 mW/cm^2, and either 15 or 30 min (total fluences of 59 and 118 J/cm^2, respectively). As the plating technique employed to determine % survival did not allow for detection of survival rates of <0.001%, data points below the detection limit were set to 0.001% survival for graphing purposes. The shaded areas correspond to undetectable cell survival with the assay employed. In the cases where error bars cannot be visualized, the error bars themselves were smaller than the marker employed in the plot.

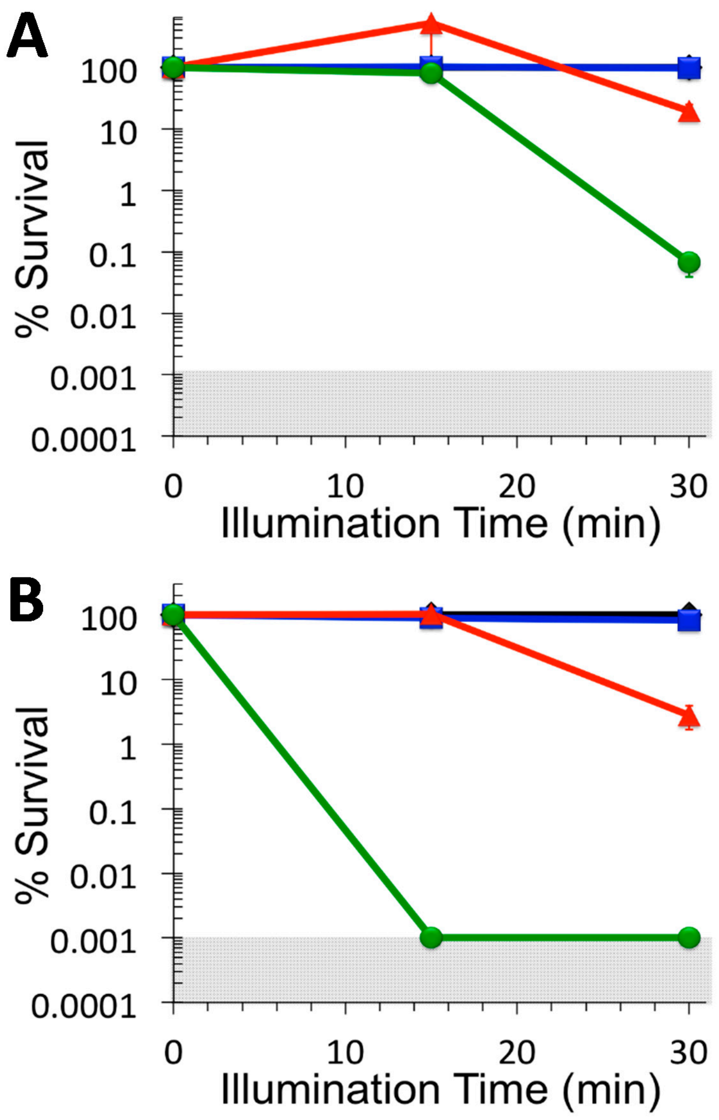

Figure S7. Photodynamic inactivation of *Candida glabrata* ATCC-15545 as a function of photosensitizer concentration and illumination time for A) methylene blue and B) TMPyP. Displayed is the % survival of the dark control (♦) and the light treated samples at 0.1 µM (■), 1 µM (▲), and 5 µM (●). Illumination conditions were as follows: 400–700 nm, 65 ± 5 mW/cm^2, and either 15 or 30 min (total fluences of 59 and 118 J/cm^2, respectively). As the plating technique employed to determine % survival did not allow for detection of survival rates of <0.001%, data points below the detection limit were set to 0.001% survival for graphing purposes. The shaded areas correspond to undetectable cell survival with the assay employed. In the cases where error bars cannot be visualized, the error bars themselves were smaller than the marker employed in the plot.

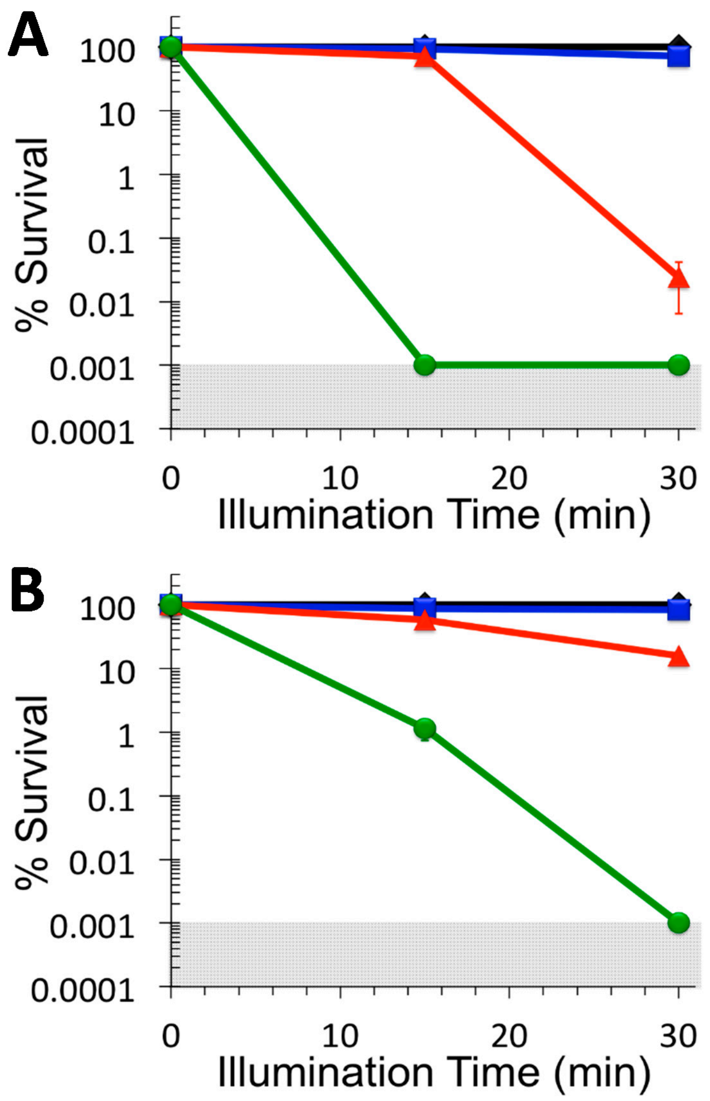

Figure S8. Photodynamic inactivation of *Cryptococcus neoformans* ATCC-64538 as a function of photosensitizer concentration and illumination time for (A) methylene blue and (B) TMPyP. Displayed is the % survival of the dark control (♦) and the light treated samples at 0.1 μM (■), 1 μM (▲), and 5 μM (●). Illumination conditions were as follows: 400–700 nm, 65 ± 5 mW/cm^2, and either 15 or 30 min (total fluences of 59 and 118 J/cm^2, respectively). As the plating technique employed to determine % survival did not allow for detection of survival rates of <0.001%, data points below the detection limit were set to 0.001% survival for graphing purposes. The shaded areas correspond to undetectable cell survival with the assay employed. In the cases where error bars cannot be visualized, the error bars themselves were smaller than the marker employed in the plot.

References

1. Fornili, S.L.; Sgroi, G.; Izzo, V. Solvent isotope effect in the monomer-dimer equilibrium of methylene blue. *J. Chem. Soc. Faraday Trans. 1* **1981**, *77*, 3049–3053.

2. Kim, J.O.; Lee, Y.A.; Yun, B.H.; Han, S.W.; Kwag, S.T.; Kim, S.K. Binding of *meso*-Tetrakis(*N*-methylpyridinium-4-yl)porphyrin to AT Oligomers: Effect of Chain Length and the Location of the Porphyrin Stacking. *Biophys. J.* **2004**, *86*, 1012–1017.
3. Caruso, E.; Banfi, S.; Barbieri, P.; Leva, B.; Orlandi, V.T. Synthesis and antibacterial activity of novel cationic BODIPY photosensitizers. *J. Photochem. Photobiol. B* **2012**, *114*, 44–51.
